# Life history mediates the trade‐offs among different components of demographic resilience

**DOI:** 10.1111/ele.14004

**Published:** 2022-03-25

**Authors:** Pol Capdevila, Iain Stott, James Cant, Maria Beger, Gwilym Rowlands, Molly Grace, Roberto Salguero‐Gómez

**Affiliations:** ^1^ Zoology Department Oxford University Oxford UK; ^2^ School of Biological Sciences University of Bristol Bristol UK; ^3^ School of Life and Environmental Sciences University of Lincoln Lincoln UK; ^4^ School of Biology Faculty of Biological Sciences University of Leeds Leeds UK; ^5^ Centre for Biodiversity and Conservation Science School of Biological Sciences University of Queensland Brisbane Australia; ^6^ Max Planck Institute for Demographic Research Rostock Germany

**Keywords:** comparative biology, conservation, disturbance, fast–slow continuum, pace of life, population collapse, recovery, resistance, stability, traits

## Abstract

Accelerating rates of biodiversity loss underscore the need to understand how species achieve resilience—the ability to resist and recover from a/biotic disturbances. Yet, the factors determining the resilience of species remain poorly understood, due to disagreements on its definition and the lack of large‐scale analyses. Here, we investigate how the life history of 910 natural populations of animals and plants predicts their intrinsic ability to be resilient. We show that demographic resilience can be achieved through different combinations of compensation, resistance and recovery after a disturbance. We demonstrate that these resilience components are highly correlated with life history traits related to the species’ pace of life and reproductive strategy. Species with longer generation times require longer recovery times post‐disturbance, whilst those with greater reproductive capacity have greater resistance and compensation. Our findings highlight the key role of life history traits to understand species resilience, improving our ability to predict how natural populations cope with disturbance regimes.

## Graphical abstract

Understanding how the resilience of species arises has become a key question in ecology and conservation. Here, we find that the demographic resilience of species is determined by three interrelated components: resistance, compensation and time of recovery. Moreover, these components of resilience are highly determined by key life history traits, such as generation time and mean reproductive output.

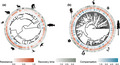

## INTRODUCTION

Preventing biodiversity loss in the face of global change is a major challenge in ecology and conservation (Folke et al., 2004; Scheffer et al., 2012). As global change accelerates (Hoegh‐Guldberg et al., 2018), species—and the services that they provide (Pecl et al., 2017)—are being lost at an unprecedented rate (Barnosky et al., 2012; Pimm et al., 2014). Still, some species can persist or even increase their abundance despite the increasingly frequent and intense disturbance events, as a consequence of global change (Antão et al., 2020; Blowes et al., 2019; van Klink et al., 2020). Such an ability to persist after a disturbance depends, to a large extent, on the species’ inherent ability to resist and recover from such events, their resilience (Capdevila, Stott, et al., 2020; Hodgson et al., 2015). Therefore, understanding what makes some species more/less resilient than others is crucial to developing effective management and conservation plans (Pressey et al., 2007). Yet, the lack of data regarding species’ natural population's responses to disturbances and robust methods to quantify resilience have hampered our understanding of the mechanisms that confer resilience to species (Hodgson et al., 2015; Ingrisch & Bahn, 2018; Willis et al., 2018).

**FIGURE 1:**
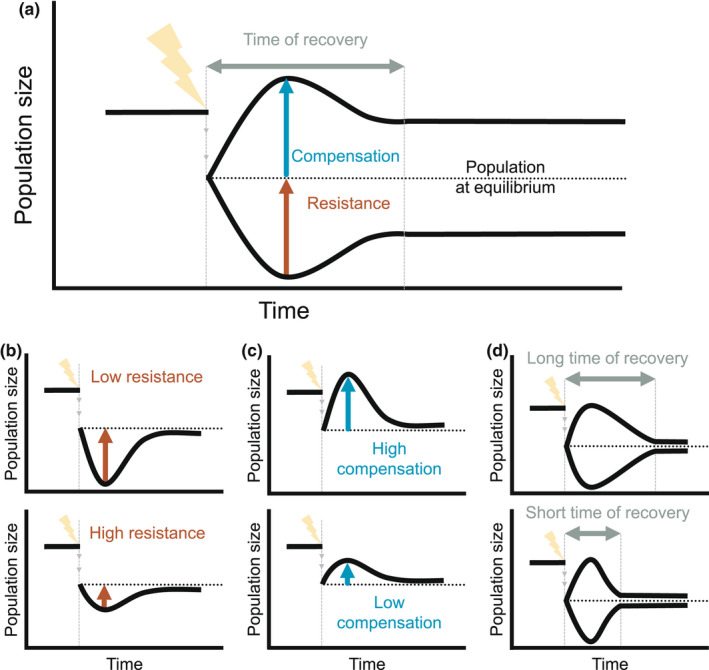
The resilience of a wild population can be quantified via three components: compensation, resistance and recovery time. (a) Decomposition of the demographic resilience components of a population affected by a punctual disturbance (lightning bolt). After a disturbance, the size and growth rate of a population may change differently according to how the population structure (i.e. the proportion of individuals at different age/stage/size in the population) is affected by the disturbance. (b) Resistance, the ability to prevent a decline in population size following a disturbance, is measured as the inverse of a population's decrease following a disturbance relative to its undisturbed conditions (i.e. with stable population structure (Stott et al., 2011)). Low resistance describes high population declines relative to a stable population. (c) Compensation, the ability to increase relative to the population size following a disturbance, is quantified as the population's increase with respect to an undisturbed population. Large increases in population size indicate high compensation, whilst small increases indicate low compensation. (d) Recovery time, the period that a population needs to reattain a stable structure after a disturbance

Understanding what factors render a species resilient requires knowledge about its population dynamics in the context of disturbances. Recent reviews suggest that studies examining the factors affecting resilience have historically focused on ecological communities or whole ecosystems rather than populations (Donohue et al., 2016; Kéfi et al., 2019). However, these studies operating at high levels of biological organisation often lack the level of detail necessary to identify the underlying processes that modulate species responses to disturbances. In contrast, demographic resilience (Capdevila, Stott, et al., 2020), i.e. the ability of a population to prevail after a disturbance, allows for a nuanced exploration of the mechanisms that confer resilience to natural populations. To quantify demographic resilience, disturbances are defined as external, punctual events that cause changes in population structure (Capdevila, Stott, et al., 2020; Stott et al., 2011), i.e., the relative proportion of individuals of different size, ages and/or stages in the life cycle of the population. Such disturbances might lead to a relative over‐ or under‐representation of individuals with high survival and/or reproduction, which ultimately will increase or decrease population size (Stott et al., 2011; Townley & Hodgson, 2008). Importantly, demographic resilience is a property of the population, as it depends on the characteristics of its life cycle and the vital rates (survival, development and reproduction) that shape its persistence (Stott et al., 2011). Demographic resilience can be captured by three key components: (1) *resistance*, the ability of a population to avoid a decrease in size after a disturbance; (2) *compensation*, the ability of a population to increase its size after a disturbance and (3) *recovery time*, the time that a population requires to recover its stable demographic structure after a disturbance event (Capdevila, Stott, et al., 2020) (Figure 1). Because these three demographic resilience components are based on vital rates common to any species (Caswell, 2001; Stearns, 1983), they can be quantified and compared among species and populations.

Finding generic relations among the three components of resilience is crucial to predicting population responses to disturbances. Correlations between resilience components implemented at the community and ecosystem levels often show complex patterns (Donohue et al., 2013; Hillebrand et al., 2018). For example, Hillebrand et al. (2018) did not find a correlation between resistance and recovery time in experimental plankton communities disturbed by reduced light availability. However, at the species level, resilience components should correlate strongly because they emerge from combinations of vital rates that are under strong selection pressures and frequently trade‐off (Stott et al., 2011). A significant correlation between two resilience components would allow us to infer one from the other. A negative correlation would indicate that the species faces a trade‐off when maximising either resilience component. Therefore, understanding the strength and direction of correlations between the components of demographic resilience may help understand and predict whether and how populations may persist despite global change.

The life history strategy of a species is likely a useful proxy to predict its demographic resilience. A species’ life history strategy summarises how energy is allocated to survival, development and reproduction throughout the lifetime of individuals to optimise their fitness (Stearns, 1992). Importantly, life history strategies can influence a species’ response to disturbances (Gamelon et al., 2014; Jelbert et al., 2019; McDonald et al., 2017; Morris et al., 2008; Stott, Franco, et al., 2010) and thus its vulnerability to extinction (Cardillo et al., 2005; Davidson et al., 2009; Fisher & Owens, 2004; Staerk et al., 2019). For instance, species with large body size, long generation times and low reproductive output (i.e. slow species, sensu Stearns, 1992) are often more vulnerable to punctual disturbances than species with small bodies, short generations and highly reproductive (Pacifici et al., 2017; Pimm et al., 2014). As such, ‘slow’ species are expected to be less demographically resilient (Salguero‐Gómez, Jones, Jongejans et al., 2016) to punctual disturbances than ‘fast’ species (i.e. species with small body sizes, short generation times and high reproductive outputs). Still, most of our understanding about the linkages between life histories and demographic resilience comes from theoretical studies using simulated data (e.g. Ezard et al., 2010; Koons et al., 2006). Besides, the few studies using empirical data have focused on only one or a few components of resilience, such as resistance or recovery (Gamelon et al., 2014; Jelbert et al., 2019; McDonald et al., 2016). Consequently, we still lack direct links between the multiple components of resilience (compensation, resistance and recovery time) and species’ life history strategies.

Here, we examine the mechanisms that confer demographic resilience to species’ populations. Specifically, we test the following hypotheses: (H1) The components of demographic resilience should be strongly related to each other, given that they result from different combinations of species’ vital rates (Stott et al., 2011); (H2a) Species with longer generation times would need longer recovery times but would have low resistance and compensation given their slow dynamics (Gamelon et al., 2014; Jelbert et al., 2019; Stott et al., 2011); (H2b) Species with high reproductive output should show high resistance and high compensation abilities, together with shorter recovery times, given their ability to quickly offset mortality events through reproductive events (Jelbert et al., 2019; Stott et al., 2011); (H3) We expect the abovementioned correlations among resilience components and with life history traits to be different between plants and animals. Plants and animals have different evolutionary histories (Graham et al., 2000; Streelman & Danley, 2003), and as a consequence, they might experience different life history trade‐offs (Healy et al., 2019; Salguero‐Gómez, Jones, Archer et al., 2016). For instance, slow‐living plant species (e.g. trees) can be highly reproductive (Stott, Franco, et al., 2010), whilst this is often not the case in slow‐living animals (e.g. whales; Fujiwara & Caswell, 2001). Because demographic resilience is tightly linked to the vital rates of the populations (Koons et al., 2006; Stott et al., 2011), we expect these two groups to show different correlation patterns among the resilience components and their life history traits.

To test the abovementioned hypotheses, we use global demographic information for 162 wild populations of 69 animal species and 748 wild populations of 232 plant species (Data S1, Table S1), from the open‐access databases COMADRE (Salguero‐Gómez, Jones, Jongejans et al., 2016) and COMPADRE (Salguero‐Gómez et al., 2015), respectively. We also couple these demographic data with phylogenetic information for animals (Michonneau et al., 2016) and plants (Jin & Qian, 2019) separately, to account for the lack of independence between the species studied (Blomberg & Garland, 2002). To establish links between the life history strategy of each species and their demographic resilience, we use these demographic data to estimate key life history traits: generation time (i.e., mean age of reproductive individuals in the population) as an indicator of a species’ pace of life (Gaillard et al., 2005; Staerk et al., 2019) and mean reproductive output (mean number of recruits produced during the mean life expectancy of an individual in the population) as an indicator of a species’ reproductive strategy (Salguero‐Gómez, Jones, Archer et al., 2016). We use multilevel Bayesian models to test for correlations between the components of demographic resilience, and with species’ life history strategies, and the potential of phylogenetic relationships influencing the observed patterns.

## MATERIAL AND METHODS

### Data selection

To calculate animal and plant demographic resilience and life history traits, we used matrix population models (MPMs) from the COMADRE Animal Matrix Database version 4.21.8.0 (Salguero‐Gómez, Jones, Jongejans et al., 2016) and COMPADRE Plant Matrix Database version 6.21.8.0 (Salguero‐Gómez et al., 2015). These databases contain demographic data compiled as age‐, size‐ or developmental stage‐structured MPMs (Caswell, 2001) for over 1000 plant and animal species. MPMs are mathematical representations of a species’ life cycle (Caswell, 2001), where each entry of the projection matrix ***A*** is a product of the vital rates of each stage, size or age of the species. That is, each column of matrix ***A*** contains all contributions by an average individual in a particular class at time *t*, whilst each row contains all contributions towards the number of individuals in a particular class at time *t*+1:

(1) $$n\left(t+1\right)=\;A\times n\left(t\right),$$

where ***n*** represents the population vector of abundances based on stages, sizes and/or ages. Where the dominant eigenvalue of ***A***, *λ*_1_, represents the asymptotically stable population growth rate (Caswell, 2001) and the right eigenvector ***w*** represents the stable stage structure, that is the relative frequency of individuals in each stage, size and/or age at stationary equilibrium.

To estimate the demographic resilience components and life history traits, we used the individual MPMs for each available population, after a set of selection criteria (below). When the individual population model ***A*** was not available, we used the mean matrices (e.g. average all ***A*** among different years within a given population). To ensure capturing the natural dynamics of each population, we only included matrices parameterised from non‐captive populations in unmanipulated (i.e. control) conditions. We also only included described annual dynamics (not seasonal or multiannual) to allow fair comparisons among the different species and populations based on annual time units of population responses to disturbance (e.g. recovery time in years) and life history traits (e.g. generation time in years). To ensure that each MPM represented a complete life cycle, we only included those that were irreducible, primitive and ergodic (Stott, Townley, et al., 2010). The resulting dataset comprised demographic information for 162 populations from 69 species of animals from the COMADRE database (Salguero‐Gómez, Jones, Jongejans et al., 2016), including 15 populations of Actinopterygii, 30 birds, 80 mammals and 37 reptiles (Table S1). The plant data comprised 748 populations from 232 species of plants from COMPADRE database (Salguero‐Gómez et al., 2015), including 744 populations of angiosperms and four gymnosperms (Table S1).

### Demographic resilience

The ***A*** matrix is also assumed to have asymptotic dynamics. That is, when using these models, it is often assumed that the population is at its stable stage distribution ***w*** (Caswell, 2001). However, disturbances can change a population's size and structure, displacing it away from equilibrium structure. Such alterations in population size and structure result in short‐term dynamics that can differ from asymptotic dynamics (Stott et al., 2011), resulting in either faster or slower growth than that at equilibrium (amplification and attenuation, respectively; Stott et al., 2011). These transient dynamics represent the intrinsic ability of populations to respond to disturbances, i.e. their demographic resilience (Capdevila, Stott, et al., 2020).

From each selected MPM, we estimated three components of resilience: compensation, resistance and recovery time (Capdevila, Stott, et al., 2020). The single time step measures of resistance and compensation we used represent transient growth once the stationary stable component of population growth or decline (*λ_1_*) has been factored out. Thus, our measures include only the (non‐stable) component of resilience that is linked to disturbance and enable fair comparisons among species and populations with different rates of stationary stable growth (Stott et al., 2011). We achieved this by ‘normalising’ the ***A*** matrices, which involves scaling each element of ***A*** by *λ_1_*, resulting in ***Â***, where *λ_1_(* ***Â*** *)* = 1. Using ***Â*** in calculations yields equivalent measures to the ratio of total growth as predicted by projecting the population using Equation 1, to the stationary stable growth as predicted by *λ_1_*, the dominant eigenvalue of ***A*** (a ratio rather than a difference is used as population growth rates are geometric). This normalisation can also be interpreted as ‘relative growth/decline’, describing how much faster or slower a population grows, relative to how fast it grows when stationarily stable (i.e., the relative number of individuals in each stage does not change; Stott, Franco, et al., 2010; Stott Townley et al., 2011). Therefore, the normalisation of the matrices allows us to measure only the component of a population dynamic that depends on the disturbance, measuring growth relative to a stable dynamic that would exist whether the population is disturbed or not. Without the normalisation of the matrix, it would not be possible to disentangle whether a population grows fast because it has a fast stationary stable growth (independent of resilience to disturbance), or a high resilience to disturbance (independent of stable growth).

Compensation is estimated as the fastest population growth that can be realised in the first time step after disturbance (first time step attenuation ${\bar{\rho }}_{1}$_,_ *sensu* Townley & Hodgson, 2008; Stott et al., 2011), which can be calculated as:

(2) $${\bar{\rho }}_{1}=\|\hat{A}{\|}_{1},$$

Resistance was estimated as the lowest population density that can be reached in the first time step after disturbance (${\underline{\rho }}_{1}$) (Stott et al., 2011; Townley & Hodgson, 2008) and was calculated as:

(3) $${\underline{\rho }}_{1}=minCS\left(\hat{A}\right),$$

where *minCS* is the minimum column sum of a matrix. Equation 3 values vary from 0 to 1, where 0 means high resistance and 1 means low resistance. To facilitate the interpretation of our resistance estimate, we corrected Equation 3 by subtracting from 1 (1‐${\underline{\rho }}_{1}$) so that values closer to 1 correspond to high resistance and 0 to low resistance.

Recovery time (*t_x_*) was estimated as the time required for the contribution of the dominant eigenvalue (*λ_1_*) to become *x* times as great as that of the subdominant eigenvalue (*λ_2_*), following:

(4) $$t_{x}=\log(\lambda_{1}/\left|\right|\lambda_{2}\left||\right)/\log\left(x\right),$$

where *x* is set at 10. A value of *x* = 10 indicates that the return time is defined by the time point at which the dominant eigenvalue has ten times the influence of the subdominant eigenvalue. In the calculation of recovery time, *x* acts as a scalar to map the damping ratio (*λ_1_* / *|| λ_2_* ||) onto the time scale. Therefore, the choice of *x* has no bearing on the relative values of recovery times between species.

All the transients were calculated using the *popdemo* R package (Stott et al., 2012).

### Life history traits and vital rates

From each MPM ***A*** (without normalisation), we calculated two key life history traits, generation time and mean reproductive output. Generation time represents the mean age of reproductive individuals in the population. Generation time (*T*) was calculated using the function *generation time* from *popbio* R package (Stubben & Milligan, 2007), which estimates *T* as:

(5) $$T=\log\left(R_{0}\right)/\log\left(\lambda_{1}\right)),$$

where *R_0_* is the net reproductive rate and *λ_1_* is the dominant eigenvalue of ***A***.

To calculate the mean reproductive output (*φ*), we sum the total sexual reproductive output for each stage of the reproductive component of ***A*** and then weight it by each stage's relative frequency at stationary equilibrium, as defined by the stable stage distribution ***w*** (Salguero‐Gómez, Jones, Archer et al., 2016).

### Phylogenetic corrections

To account for the phylogenetic relatedness of the species in our analyses, we used phylogenies for animals and plants. The plant phylogeny was obtained using the *V*. *PhyloMaker* R package (Jin & Qian, 2019). *V*. *PhyloMaker* allows building a rooted and time‐calibrated phylogeny using a species list based on already built plant phylogenies (Smith & Brown, 2018; Zanne et al., 2014). The animal phylogeny was produced using the *datelife* R package (Sánchez‐Reyes & O’Meara, 2019), a platform that uses publicly accessible phylogenetic source data to build a chronogram—rooted and time‐calibrated tree—given an input phylogeny that we sourced from the Open Tree of Life (Hinchliff et al., 2015; Michonneau et al., 2016). In some cases, for both plant and animal phylogenies, we detected polytomies (i.e. >2 species with the same direct ancestor), which can interfere in our phylogenetic signal analyses (Revell, 2012). Polytomies were resolved using the function *multi2di* from *ape* package (Paradis et al., 2004). Briefly, this approach transforms polytomies into a series of random dichotomies with one or several branches of length very close to 0.

### Statistical analyses

We used different Bayesian multilevel models with varying structures to address the different research questions tackled here. For all the analyses, we log‐transformed (natural logarithm) resistance, compensation, recovery time, generation time and mean reproductive output and z‐scaled their values before performing the respective analyses. All the models were fitted using the *brms* package v2.1.0 (Bürkner, 2017) in R v4.0.0 (R Core Team, 2020) and run for 8000 iterations, with a warm‐up of 800 iterations. Convergence was assessed visually by examining trace plots and using Rhat values (the ratio of the effective sample size to the overall number of iterations, with values close to one indicating convergence).

To explore the influence of the evolutionary history in determining the patterns of variation of the demographic resilience components, we modelled compensation, resistance and recovery time across phylogenetic relatedness. We fitted one model per demographic resilience components using multilevel Bayesian models without fixed effects and including the variance‐covariance matrix of the phylogeny and species as random effects. To account for the potential effects of the MPM state variable (size/age/stage‐based) on the demographic resilience components’ estimates (as suggested in Kendall et al., 2019), we included the model state variable as a random factor. We considered age‐ and stage‐based matrices as a single matrix type, and when size and stage were combined, we considered them as stage‐based. We estimated the phylogenetic signal by calculating the explained variance of the random effects (phylogeny and population) in the posterior distributions of the models. A distribution pushed up against 0 indicates a lack of phylogenetic signal, given that the variance explained is bounded by 0 and only has positive values. We set weakly informed priors:

(6) $$y_{i,j}∼\mathrm{Normal}\left(\mu_{i,j,k},\;\sigma^{2}\right),$$

(7) $$\mu_{i,j,k}=\beta_{0}+\beta_{0i}+\beta_{0j}+\beta_{0k},$$

(8) $$\beta_{0}∼\mathrm{Normal}\left(0,10\right),$$

(9) $$\sigma^{2}∼\;\mathrm{Exponential}\left(1\right),$$

where $y_{i,j}$ is the estimate for compensation, resistance and recovery time for the *i^th^* species, *j^th^* phylogenetic distance and *k^th^* model state variable and is given by a normal distribution with mean *μ* and variance $\sigma^{2}$. $\beta_{0}$ is the global intercept; $\beta_{0i}$, $\beta_{0j}$ and $\beta_{0k}$ are the population‐level, phylogenetic‐level and the model state variable departure from $\beta_{0}$, respectively.

To estimate the correlation among the components of demographic resilience, we fitted a multivariate multilevel model with compensation, resistance and recovery time as response variables and without predictors (McElreath, 2020). Then, given that there were no predictors in the model, the residual correlations represented the correlation between compensation, resistance and recovery time. The residual correlation resembles classical Pearson's correlations with values varying from −1 to +1, indicating negative to positive correlations, respectively, and values close to 0 indicating lack of correlation. To account for the lack of independence between the species in our analyses, we used the variance‐covariance metric as a random factor (see above). We also accounted for the potential effects of the model state variable (size/stage‐based) on the demographic resilience components (as suggested in Kendall et al., 2019) by including it as a random factor. We used a Student's t‐distribution as the likelihood rather than a normal distribution. We used a Student's t‐distribution as the likelihood because this distribution is less sensitive to multivariate outliers (Kruschke, 2014). As priors, we used:

(10) $$y_{j}∼\text{Student'}s\;t\left(\mu_{i,j,k},v\right),$$

(11) $$\mu_{j}=\beta_{0}+\;\beta_{0i}+\beta_{0j}+\beta_{0k},$$

(12) $$\beta_{0}∼\mathrm{Normal}\left(0,10\right),$$

(13) $$\nu ∼\mathrm{Gamma}\left(2,0.1\right),$$

where $y_{j}$ is the estimate for compensation, resistance and recovery time for the *i^th^* population, *j^th^* phylogenetic distance and *k^th^* model state variable and is given by a Student's t distribution with mean *μ* and the degrees of freedom ν. $\beta_{0}$ is the global intercept, $\beta_{0i}$, $\beta_{0j}$ and $\beta_{0k}$ are the population‐level, phylogenetic‐level and the model state variable departure from $\beta_{0}$, respectively.

Finally, to estimate the relationship between the components of demographic resilience and the life history strategy of the species, we performed a multivariate multilevel model using compensation, resistance and recovery time as response variables and generation time, mean reproductive output and their interaction as predictors. We also used matrix dimension as a covariate to control for potential confounding effects of the size of the matrix and the resilience component (Stott, Franco, et al., 2010). We also accounted for the lack of independence between the species analysed by incorporating the variance‐covariance matrix derived from the phylogeny in the model as a random factor. To depict further differences between the studied species not accounted for by the phylogeny, we added population as a random factor. Here, we also accounted for the potential effects of the model state variable (size/stage‐based) by including it as a random factor. In addition, due to the different scales of the life history traits and the resilience components, we z‐scaled all the variables. For these models, we used weakly regularising normally distributed priors for the global intercept and slope:

(14) $$y_{i,j,k}∼\mathrm{Normal}\left(\mu_{i,j,k},\;\sigma^{2}\right),$$

(15) $$\begin{array}{c} \mu_{i,j}=\beta_{0}+\beta_{0i}+\beta_{0j}+\beta_{0k}+\beta_{G}+\beta_{\mathrm{Gi}}+\beta_{\mathrm{Gj}}\\ \;+\beta_{\mathrm{Gk}}+\beta_{R}+\beta_{\mathrm{Ri}}+\beta_{\mathrm{Rj}}+\beta_{\mathrm{Rk}}+\beta_{G}:\beta_{R}\\ \;+\beta_{\mathrm{Gi}}:\beta_{\mathrm{Ri}}+\beta_{\mathrm{Gj}}:\beta_{\mathrm{Rj}}+\beta_{\mathrm{Gk}}:\beta_{\mathrm{Rk}}+\beta_{D}+\beta_{\mathrm{Di}}\\ \;+\beta_{\mathrm{Dj}}+\beta_{\mathrm{Dk}}, \end{array}$$

(16) $$\beta_{0}∼\;\mathrm{Normal}\left(0,1\right),$$

(17) $$\beta ∼\;\mathrm{Normal}\left(0,10\right),$$

(18) $$\sigma^{2}∼\mathrm{Normal}\left(0,1\right),$$

where $y_{i,j}$ is the estimate for compensation, resistance and recovery time for the *i^th^* population, for the *j^th^* phylogenetic distance, and *k^th^* model state variable. $\beta_{0}$ is the global intercept; $\beta_{0i}$, $\beta_{0j}$ and $\beta_{0k}$ are the population‐level, phylogenetic‐level and the model state variable departure from $\beta_{0}$, respectively. $\beta_{G}$, $\beta_{R}$ and $\beta_{D}$ represent the effects of generation time, mean reproductive output and matrix dimension, respectively.

## RESULTS

### Evolutionary history explains more variation in the demographic resilience of animals than in plants

**FIGURE 2:**
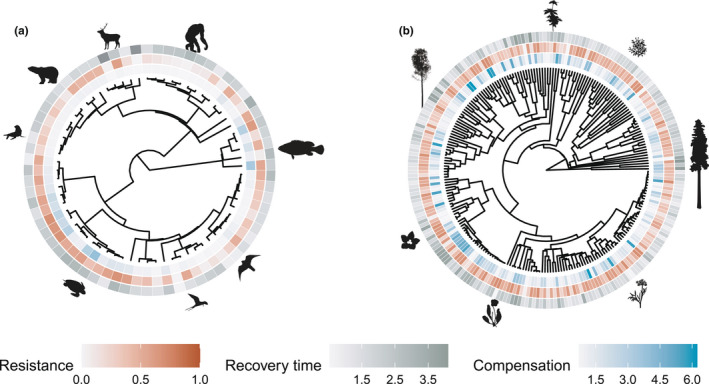
Evolutionary history explains a higher degree of variability of the demographic resilience in animals than in plants. Patterns of variation of demographic compensation, resistance and recovery time (Figure 1) for the examined 162 populations of 69 animal species and 748 populations of 232 plant species. The inward ring represents resistance, middle ring compensation and outer ring recovery time. Evolutionary history explains a greater amount of variability of demographic resilience in animals (a) than in plants (b). Values showed in each panel represent the mean values of compensation, resistance and recovery time per species. (a) In animals, the phylogenetic signal was stronger for compensation (0.63 ± 0.18, mean ± SE), than for resistance (0.48 ± 0.26) and recovery time (0.41 ± 0.21). Silhouettes represent, from the top in a clockwise direction, chimpanzee (*Pan troglodytes*), red grouper (*Epinephelus morio*), peregrine falcon (*Falco peregrinus*), common tern (*Sterna hirundo*), green sea turtle (*Chelonia mydas*), California sea lion (*Zalophus californianus*), polar bear (*Ursus maritimus*) and red deer (*Cervus elaphus*). (b) In plants, compensation (0.04 ± 0.05) and resistance (0.02 ± 0.04) show a weak phylogenetic signal, whilst recovery time had a stronger phylogenetic signal (0.66 ± 0.08). Silhouettes represent, from the top in a clockwise direction, woodland geranium (*Geranium sylvaticum*), wild plantain (*Heliconia acuminata*), white Cypress‐pine (*Callitris columellaris*), alpine sea holly (*Eryngium alpinum*), purple pitcher plant (*Sarracenia purpurea*), Douglas's catchfly (*Silene douglasii*) and grey alder (*Alnus incana*). Silhouettes’ source: phylopic.org

The evolutionary history of the examined species plays an important role in explaining the variation of the demographic resilience of animals, but not in plants (Figure 2). The studied animal species show a strong phylogenetic signal for all three components of demographic resilience (Figure 2a), but with a stronger role in the variation of compensation (0.55 ± 0.22, mean ± SE), than in resistance (0.39 ± 0.27) or over recovery time (0.35 ± 0.22). In contrast, evolutionary history plays a minor role in explaining the variation of plant demographic resilience (Figure 2b). In particular, compensation (0.09 ± 0.09) and resistance (0.02 ± 0.04) show a weak phylogenetic signal, compared to recovery time (0.61 ± 0.15). Overall, these results suggest that whilst resistance and compensation are highly influenced by the evolutionary history in animals, they are less evolutionarily constrained in plants.

### Demographic resilience components trade‐off strongly to shape species resilience

**FIGURE 3:**
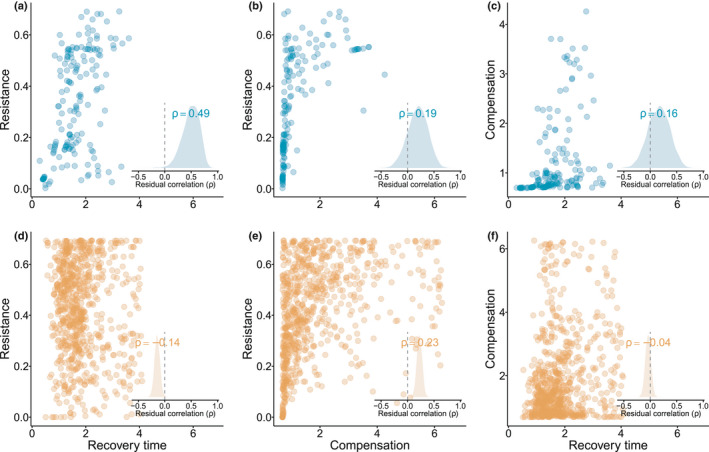
The components of demographic resilience correlate differently for plants than for animals. Correlations between the components of resilience, (a, d) resistance *vs*. recovery time, (b, e) resistance *vs*. compensation and (c, f) recovery time *vs*. compensation for 162 populations of 69 animal species (a–c) and 748 populations of 232 plant species (d–f). Insets show the distribution of the residual correlations between the components of resilience, where *ρ* represents the mean value of the distribution. Positive values of *ρ* indicate a positive correlation between components, and negative values represent a trade‐off. The correlation between resistance and recovery time is (a) positive for animals but (d) negative for plants. Resistance and compensation are positively correlated in both (b) animals and (e) plants. Recovery time and compensation are (c) slightly positively correlated in animals and (f) slight negatively correlated in plants. The residual correlations were estimated by fitting a multivariate multilevel Bayesian model using compensation, resistance and recovery time as the response variable and with no predictors (see Methods)

Our results suggest the existence of trade‐offs among the components of demographic resilience. Demographic resilience in our examined species emerges either by withstanding disturbances through resistance and compensation or by minimising recovery time after a disturbance (Figure 3). The residual correlations reveal a positive value between resistance and recovery time in animals (Figure 3a). The more resistant to a disturbance an animal is, the longer it needs to recover from the disturbance that pushes its population structure away from its stationary equilibrium. In contrast, resistance and recovery time are negatively correlated in our studied plant species (Figure 3a, d). Resistance and compensation are also positively correlated in both plants and animals (Figure 3b, e), although the correlation is weaker for the latter. A positive correlation between resistance and compensation means that species with a high ability to remain stable after a disturbance also have a high ability to compensate after it. Finally, compensation and recovery time are negatively correlated in plants and positively correlated in animals, albeit with high uncertainty (Figure 3c, f).

### Life history traits predict demographic resilience

**FIGURE 4:**
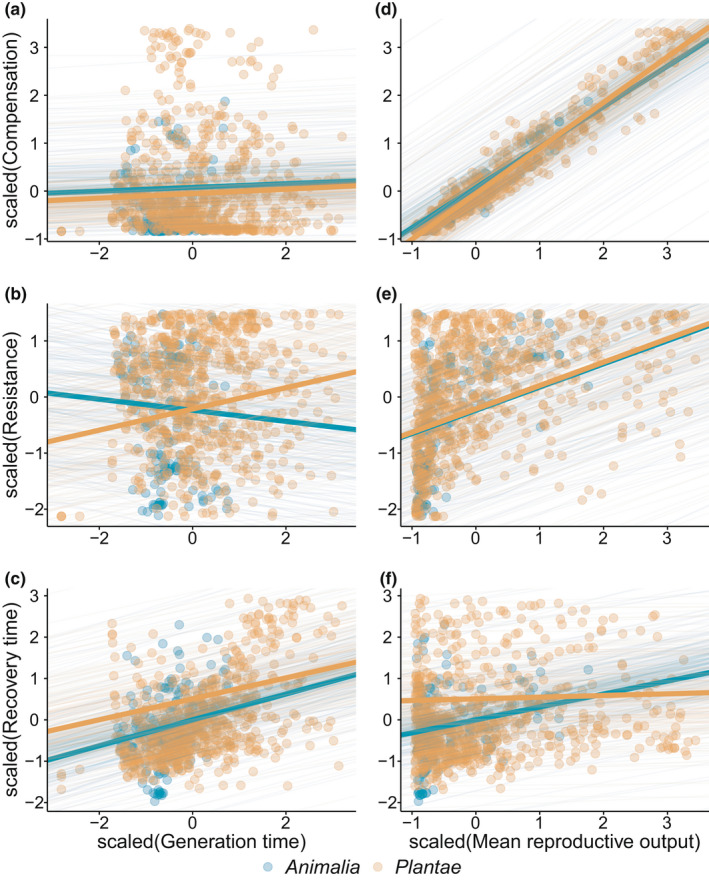
The three components of demographic resilience—resistance, compensation and recovery time—strongly correlate with two key species life history traits: generation time and mean reproductive output. (a–c) display the correlations of (a) compensation, (b) resistance and (c) recovery time, with generation time. (d–f) display the correlations of (d) compensation, (e) resistance and (f) recovery time, with mean reproductive output. Here, we show the correlations between the scaled values of the demographic resilience components of resistance, compensation and recovery time with the scaled values of generation time and reproductive output of 162 populations of 69 animal species (blue) and 748 populations of 232 plant species (orange). Lines represent the predictions from the multilevel Bayesian models (Table S2), where thin lines correspond to the predictions drawn from each of the 250 posterior samples of the model, and the thick line represents the mean outcome of the model

The components of demographic resilience are highly correlated with species’ life history traits (Figure 4). Generation time is tightly linked to resistance and recovery time, but it has no clear association with compensation (Figure 4a–c, Table S2). However, key differences do occur between plants and animals. Whilst in both groups, generation time is positively correlated with recovery time (Figure 4c, Table S2), generation time is negatively correlated with resistance in animals, but positively in plants.

The mean reproductive output of a species is a strong predictor of its components of demographic resilience (Figure 4d–f, Table S2). Here again, though, plants and animals employ slightly contrasting strategies to achieve resilience. Reproductive output is positively correlated with the resistance and compensation of both animals and plants (Figure 4d–e, Table S2). However, reproductive output is positively correlated with the recovery time of our studied animals but lacks a clear relationship with the recovery time of plants. Also, our results suggest that being highly reproductive trades‐off with longer periods of recovery, at least in the examined animal species. The interactive effects of generation time and reproductive output with the components of demographic resilience are consistent across animals and plants, with both kingdoms showing a positive interaction for compensation (Table S2), but a non‐significant effect on resistance or recovery time. Most of these patterns do not arise when using randomly simulated MPMs, with the exception of compensation and mean reproductive output (Figure S1). The similar correlation between compensation and mean reproductive output in simulated MPMs vs. natural populations suggests that they are exerted to mathematical constraints rather than being biologically meaningful. However, all the other combinations of life history traits and resilience components rendered credibly different correlations between the simulated MPMs vs. natural populations (Figure S1). Therefore, these results suggest that the other correlations among the resilience components and life history traits are not a mathematical by‐product.

## DISCUSSION

In this study, we provide empirical support that multicellular organisms can achieve demographic resilience through different combinations of compensation, resistance and recovery time. We show that these combinations of demographic resilience are largely determined by the life history strategy of each species. We also show that the evolutionary history of species plays an important role in animal but not plant demographic resilience. Indeed, a greater deal of evolutionary plasticity in life history strategies of plants has been previously reported compared to animals (Healy et al., 2019; Paniw et al., 2018). This plasticity in plant life histories (Salguero‐Gómez, Jones, Archer et al., 2016) could explain the lack of phylogenetic signal in their demographic resilience components. Incorporating environmental stochasticity and genetic data would also allow exploring the role of plasticity in the evolution of demographic resilience in further detail (Coulson et al., 2021). However, it also must be noted that plants and vertebrates have rather distinct evolutionary histories (Graham et al., 2000; Streelman & Danley, 2003), making their comparisons somewhat challenging to interpret. In addition, because animals were less well represented than plants in our dataset, the phylogenetic signal of the former could have been biased by the lower representation of different taxonomic groups (Blomberg & Garland, 2002). Whilst exploring in detail the evolutionary patterns of the demographic resilience components was out of the scope of this study, to address this question, future works should have a better representation of plant and animal taxonomic groups.

We provide evidence that the components of demographic resilience are correlated, a key finding that contrasts with studies focusing on communities or ecosystems suggesting no direct correlation (Hillebrand & Kunze, 2020; Kéfi et al., 2019; Radchuk et al., 2019). Such a discrepancy likely arises due to the different biological levels at which the components of resilience have been studied up to date (Hillebrand & Kunze, 2020; Kéfi et al., 2019). Indeed, studies focusing on physiological responses report a robust correlation between resistance and recovery time (Li et al., 2020; Lloret et al., 2011). Given that our approach is focused on intrinsic population properties (Capdevila, Stott, et al., 2020; Stott et al., 2011), each of the demographic resilient components is ultimately the result of a combination of their vital rates, such as survival and reproduction (Capdevila, Stott, et al., 2020; Stott, Franco, et al., 2010; Townley & Hodgson, 2008). Supporting this rationale, we show that both compensation and resistance are highly positively correlated with species’ mean reproductive output (Figure 4). These strong correlations between the components of demographic resilience could be the result of trade‐offs between species’ vital rates (Salguero‐Gómez, Jones, Archer et al., 2016; Stearns, 1992). However, our supplementary analyses caution against these conclusions, given that similar correlations are found in simulated matrices (Figure S1), suggesting that these might potentially be a result of mathematical constraints.

The correlation between resistance and recovery time and between compensation and recovery has opposite but consistent directions in animals and plants. In animals, these relationships are positive, whereas in plants they imply trade‐offs (negative). Given that compensation and resistance represent population size increase and decrease after disturbance (Capdevila, Stott, et al., 2020; Stott et al., 2011), respectively, one would expect populations with a greater ability to compensate (high increase) or resist (lower decline) disturbances to also recover faster. However, it is known that even if populations increase in size quickly after a disturbance or avoid declines in population size, their dynamics will oscillate until they reach their stable structure (Neubert & Caswell, 1997; Stott et al., 2011), that is, the structure previous to a disturbance here. During this transient period, the population has not yet recovered from the disturbance, as the population might experience further increases or decreases in size, which ultimately can lead to local extinction (Stott et al., 2011; Townley & Hodgson, 2008). Therefore, even if a population can compensate or resist a disturbance, it might take a long period of time to recover from its effects, no matter how small.

Differences in the relationships among the resilience components in animals and plants have likely emerged from key differences in the evolution of the life history strategies of these two kingdoms. Generation time is linked to the pace of life of species, with longer generation times being associated with slower paces of life (Gaillard et al., 1989, 2005). However, generation time and mean reproductive output are coordinated differently in plants than in animals. Animals with long generation times usually show low reproductive outputs (Bielby et al., 2007; Gaillard et al., 1989), whilst in plants (but also some animal groups, such as corals or fishes), species with long generation times can be highly reproductive (Capdevila, Beger, et al., 2020; Jones et al., 2014; Salguero‐Gómez, Jones, Archer et al., 2016). Because there is a tight link between resistance and the mean reproductive output (Figure 4), it is then not surprising to find divergent relationships between resistance and generation time for both kingdoms.

Our simulations suggest that the correlations between mean reproductive output and compensation should be interpreted with caution. Because in our study, we calculate the resilience components and the life history traits from the same MPMs, the correlations could be mathematical artefacts rather than due to biological processes. Therefore, we generated random MPMs to remove the biological constraints caused by life history trade‐offs or the evolutionary history of the species (Stearns, 1992). The correlations between the demographic resilience components and the life history traits derived from the random MPMs represent the correlations we would expect due to mathematical constraints rather than biological processes. Randomly generated MPMs show similar relationships between compensation and mean reproductive output than for our natural populations of plants and animals (Figure S1). The similarity among empirical and simulated data suggests that compensation could be mathematically, rather than biologically, linked to mean reproductive output. Despite these results, there are clear links between compensation and key biological processes. For example, compensation has been linked to the invasiveness of plants (Jelbert et al., 2019) or the ability of coral populations to persist in unstable environmental conditions (Cant et al., 2021). Thus, although our results must be carefully interpreted, we argue that compensation is still linked with the life history strategy of species, though the exact mechanism deserves further exploration. In addition, some empirical correlations show the same sign as the ones from random matrices but with lower absolute values (e.g. resistance *vs*. reproductive output; Figure S1). These results indicate that these correlations are constrained by the life history of the species (e.g. trade‐offs, evolutionary history; Stearns, 1992), limiting their absolute value.

To further explore potentially spurious associations between demographic resilience and species life histories, we repeated our analyses with life history traits from independent databases (TRY, Kattge et al., 2020; Amniote, Myhrvold et al., 2015). Animal body weight and plant height are strongly and positively correlated with generation time in plants and animals (Gaillard et al., 1989; Salguero‐Gómez, Jones, Archer et al., 2016). Using a subsample from our dataset from COMADRE and COMPADRE for which body weight and height information is available in TRY (Kattge et al., 2020) and Amniote (Myhrvold et al., 2015) databases (88.89% of animal and 44.25% plant populations), we observe similar correlations as those for generation time in animals (Figure S2a–c). However, these patterns are less clear for plant height (one of the few currently available proxies for plant body size at a macroecological scale—Figure S2a–c) or plant growth form (Figure S3, another proxy for plant life history strategies; Raunkiær, 1934; Salguero‐Gómez, Jones, Archer et al., 2016). These results confirm our previous findings that a slow life history strategy results in lower demographic resilience in animals, but not necessarily in plants. Indeed, plants attain alternate resilience strategies depending on their life history strategies: slower plant species do so via greater resistance, whilst faster plant species attain resilience via shorter recovery times.

To date, much of population ecology and demographic research has focused on how increasing environmentally driven variability of vital rates impacts the long‐term viability of populations (Boyce et al., 2006; McDonald et al., 2017; Morris et al., 2008) (but see Field et al., 2019; McDonald et al., 2016; Stott, Franco, et al., 2010). This research has led to the general consensus that species with slow life history strategies tend to be buffered from increased environmental variation (McDonald et al., 2017; Morris et al., 2008, 2011; Sæther et al., 1996). However, this consensus contrasts with the fact that animals with slower paces of life are typically more threatened than species with faster paces of life (Cardillo et al., 2005; Carmona et al., 2021). Although in our analyses we do not find strong links between the conservation status of a species and its demographic resilience (Figure S4), our results suggest that animals with slow life history strategies often do poorly with disturbances. Therefore, even if slow‐paced organisms are buffered against environmental stochasticity (Morris et al., 2011), they may still be vulnerable to disturbance events caused by other global change agents.

The resilience of a species is the outcome of multiple factors and accounting for all of them can be challenging. Our approach evaluates the potential responses of species, based on their intrinsic demographic capabilities (Capdevila, Stott, et al., 2020; Stott et al., 2011), to disturbances using the maximum values that a population can increase or decrease after a disturbance. However, the intensity, frequency, duration and temporal autocorrelation of a disturbance regime can all modify the strength and direction of the relationships among components of resilience in ecological communities (Donohue et al., 2013). Since ecosystems are currently exposed to numerous concurrent disturbances (Díaz et al., 2019), future studies should explore how the interaction between disturbances affects resilience and the correlation among its components (Donohue et al., 2013). Also, our approach does not explicitly consider density dependence, which is known to shape population responses to disturbances (Paniw et al., 2019). Most importantly, we define disturbance as a sudden event impacting the structure of the population (Capdevila, Stott, et al., 2020; Stott et al., 2011); however, perturbations can also alter the vital rates of a population (e.g. Capdevila et al., 2019; Jenouvrier et al., 2014). Changes in the vital rates will alter the stable structure of the population, generating discrepancies between the actual population structure and the stable structure. In these cases, it is also possible to measure emerging transient dynamics and resilience components (Field et al., 2019). However, the lack of large volumes of demographic data incorporating density dependence and perturbation information is currently an important barrier for comparative studies, such as the present one.

As global change advances, species are being lost at an unprecedented rate (Barnosky et al., 2011; Díaz et al., 2019), alongside the services that they provide (Díaz et al., 2019). Here, we show that the resilience of natural populations emerges from different combinations of compensation, resistance and recovery time for animals than for plants. We also demonstrate that life history strategies of species strongly determine their demographic resilience, with important differences among plants and animals. Incorporating knowledge of a species’ life history is critical to predicting how its populations may respond to disturbances. Understanding how species achieve resilience, as done here, will prove key in developing effective conservation actions.

## AUTHOR CONTRIBUTIONS

PC, RS‐G, IS and MB conceived the original idea, with contributions from IS, JC, GR and MG. PC, IS, GR and MG implemented the statistical analysis. PC, RS‐G and IS wrote the initial draft. All authors contributed to the final version of the manuscript.

### PEER REVIEW

The peer review history for this article is available at https://publons.com/publon/10.1111/ele.14004.

## Supporting information

Supplementary Material

## Data Availability

Data and code supporting the results are available in the GitHub https://github.com/PolCap/DemographicResilience and in https://doi.org/10.5281/zenodo.6337559. Matrix population models are available at www.compadre‐db.org.
