# Supplementary material for: Life history mediates the trade‐offs among different components of demographic resilience
Source: Ecol Lett. 2022 Mar 25;25(6):1566–79. doi: 10.1111/ele.14004 (PMC9314072; doi:10.1111/ele.14004)
Supplement: Supplementary file 1 — Supplementary Material [file ELE-25-1566-s001.zip › ele14004-sup-0001-Supinfo.pdf]

Life history mediates the trade-offs among different components of demographic resilience

Pol Capdevila[1,2*], Iain Stott[3], James Cant[4], Maria Beger[4,5], Gwilym Rowlands[1], Molly Grace[1], Roberto Salguero-Gómez[1,5,6]

[1]Zoology Department, Oxford University, Zoology Research and Administration Building, 11a Mansfield Rd, Oxford OX1 3SZ, UK

[2]School of Biological Sciences, University of Bristol, 24 Tyndall Ave, BS8 1TQ, Bristol, UK

[3]School of Life and Environmental Sciences, University of Lincoln, Brayford Pool, Lincoln LN6 7TS, UK

[4]School of Biology, Faculty of Biological Sciences, University of Leeds, UK, LS2 9JT

[5]Centre for Biodiversity and Conservation Science, School of Biological Sciences, University of Queensland, Brisbane, 4072, Australia

[6]Max Planck Institute for Demographic Research, Konrad Zuse Straße 1, Rostock 18057, Germany

*Corresponding author: pcapdevila.pc@gmail.com

Table S1. Species with matrix population models used in our analyses to examine the life history trait correlates of demographic resilience. "Number of populations" indicates the number of populations per species used in our analyses. See full dataset Data S1 for further details, including data source and full citation.

Kingdom	Class	Species Accepted	Number of populations
**Animalia**	Actinopterygii	*Acipenser fulvescens*	10
	Actinopterygii	*Notropis photogenis*	1
	Actinopterygii	*Epinephelus morio*	1
	Actinopterygii	*Astroblepus ubidiai*	1
	Actinopterygii	*Pylodictis olivaris*	2
	Aves	*Buteo solitarius*	4
	Aves	*Gyps coprotheres*	1
	Aves	*Haliaeetus albicilla*	2
	Aves	*Milvus migrans*	1
	Aves	*Pernis apivorus*	1
	Aves	*Anser anser*	3
	Aves	*Calidris temminckii*	2
	Aves	*Centrocercus minimus*	2
	Aves	*Sterna hirundo*	2
	Aves	*Falco peregrinus*	2
	Aves	*Lagopus muta*	1
	Aves	*Anthropoides paradiseus*	1
	Aves	*Certhia americana*	1
	Aves	*Bostrychia hagedash*	1
	Aves	*Fulmarus glacialis*	1
	Aves	*Phoebastria immutabilis*	1
	Aves	*Amazona vittata*	1
	Aves	*Calyptorhynchus lathami*	2
	Aves	*Strix occidentalis*	1
	Mammalia	*Alces alces*	8
	Mammalia	*Cervus elaphus*	4
	Mammalia	*Odocoileus virginianus*	12
	Mammalia	*Ovis canadensis*	16
	Mammalia	*Canis lupus*	1
	Mammalia	*Eumetopias jubatus*	1
	Mammalia	*Halichoerus grypus*	1
	Mammalia	*Mirounga leonina*	1
	Mammalia	*Panthera pardus*	1
	Mammalia	*Phocarctos hookeri*	2
	Mammalia	*Urocyon littoralis*	2
	Mammalia	*Ursus americanus*	4
	Mammalia	*Ursus maritimus*	1

	Mammalia	*Zalophus californianus*	7
	Mammalia	*Eidolon helvum*	1
	Mammalia	*Onychogalea fraenata*	1
	Mammalia	*Brachyteles hypoxanthus*	1
	Mammalia	*Cebus capucinus*	1
	Mammalia	*Cercopithecus mitis*	1
	Mammalia	*Gorilla beringei beringei*	1
	Mammalia	*Macaca mulatta*	5
	Mammalia	*Pan troglodytes schweinfurthii*	1
	Mammalia	*Papio cynocephalus*	1
	Mammalia	*Propithecus edwardsi*	2
	Mammalia	*Propithecus verreauxi*	1
	Mammalia	*Elephas maximus*	1
	Mammalia	*Marmota flaviventris*	1
	Mammalia	*Spermophilus dauricus*	1
	Reptilia	*Crocodylus johnsoni*	1
	Reptilia	*Crocodylus johnsoni*	3
	Reptilia	*Cryptophis nigrescens*	1
	Reptilia	*Drymarchon couperi*	1
	Reptilia	*Hoplocephalus bungaroides*	1
	Reptilia	*Phrynosoma cornutum*	2
	Reptilia	*Sceloporus grammicus*	9
	Reptilia	*Xenosaurus platyceps*	3
	Reptilia	*Caretta caretta*	1
	Reptilia	*Chelodina expansa*	2
	Reptilia	*Chelonia mydas*	2
	Reptilia	*Chelydra serpentina*	1
	Reptilia	*Chrysemys picta*	2
	Reptilia	*Clemmys guttata*	1
	Reptilia	*Emydura macquarii*	2
	Reptilia	*Kinosternon integrum*	1
	Reptilia	*Kinosternon subrubrum*	3
	Reptilia	*Malaclemys terrapin*	1
**Plantae**	Liliopsida	*Arisaema serratum*	1
	Liliopsida	*Anthericum ramosum*	4
	Liliopsida	*Corallorhiza trifida*	1
	Liliopsida	*Cypripedium calceolus*	3
	Liliopsida	*Cypripedium fasciculatum*	4
	Liliopsida	*Dactylorhiza lapponica*	4
	Liliopsida	*Neotinea ustulata*	5
	Liliopsida	*Orchis purpurea*	7
	Liliopsida	*Dioscorea chouardii*	1
	Liliopsida	*Calochortus albus*	2

	Liliopsida	*Calochortus lyallii*	15
	Liliopsida	*Calochortus macrocarpus*	4
	Liliopsida	*Calochortus obispoensis*	1
	Liliopsida	*Calochortus pulchellus*	1
	Liliopsida	*Calochortus tiburonensis*	2
	Liliopsida	*Colchicum autumnale*	6
	Liliopsida	*Erythronium japonicum*	3
	Liliopsida	*Fritillaria biflora*	2
	Liliopsida	*Fritillaria meleagris*	1
	Liliopsida	*Narcissus pseudonarcissus*	2
	Liliopsida	*Trillium camschatcense*	1
	Liliopsida	*Trillium grandiflorum*	25
	Liliopsida	*Trillium ovatum*	1
	Liliopsida	*Trillium persistens*	9
	Liliopsida	*Bursera glabrifolia*	1
	Liliopsida	*Carex humilis*	1
	Liliopsida	*Danthonia sericea*	7
	Liliopsida	*Festuca eskia*	2
	Liliopsida	*Hilaria mutica*	3
	Liliopsida	*Hyparrhenia diplandra*	1
	Liliopsida	*Molinia caerulea*	5
	Liliopsida	*Poa alpina*	3
	Liliopsida	*Sporobolus heterolepis*	1
	Liliopsida	*Swallenia alexandrae*	1
	Liliopsida	*Zea diploperennis*	1
	Liliopsida	*Calathea ovandensis*	1
	Liliopsida	*Heliconia acuminata*	5
	Magnoliopsida	*Chaerophyllum aureum*	1
	Magnoliopsida	*Eryngium alpinum*	1
	Magnoliopsida	*Eryngium cuneifolium*	5
	Magnoliopsida	*Heracleum mantegazzianum*	1
	Magnoliopsida	*Laserpitium longiradium*	1
	Magnoliopsida	*Lomatium bradshawii*	9
	Magnoliopsida	*Lomatium cookii*	3
	Magnoliopsida	*Panax quinquefolius*	4
	Magnoliopsida	*Arnica angustifolia*	1
	Magnoliopsida	*Artemisia genipi*	3
	Magnoliopsida	*Carduus nutans*	4
	Magnoliopsida	*Carlina vulgaris*	1
	Magnoliopsida	*Centaurea horrida*	4
	Magnoliopsida	*Centaurea jacea*	3
	Magnoliopsida	*Cheirolophus metlesicsii*	1
	Magnoliopsida	*Cirsium acaule*	1

	Magnoliopsida	*Cirsium dissectum*	1
	Magnoliopsida	*Cirsium pannonicum*	1
	Magnoliopsida	*Cirsium perplexans*	2
	Magnoliopsida	*Cirsium pitcheri*	8
	Magnoliopsida	*Cirsium scariosum*	2
	Magnoliopsida	*Cirsium tracyi*	1
	Magnoliopsida	*Cirsium vulgare*	1
	Magnoliopsida	*Echinacea angustifolia*	3
	Magnoliopsida	*Eupatorium perfoliatum*	3
	Magnoliopsida	*Eupatorium resinosum*	2
	Magnoliopsida	*Helianthus divaricatus*	1
	Magnoliopsida	*Jurinea fontqueri*	1
	Magnoliopsida	*Liatris scariosa*	4
	Magnoliopsida	*Picris hieracioides*	1
	Magnoliopsida	*Pilosella floribunda*	1
	Magnoliopsida	*Pyrrocoma radiata*	6
	Magnoliopsida	*Santolina melidensis*	1
	Magnoliopsida	*Saussurea medusa*	1
	Magnoliopsida	*Scorzonera hispanica*	1
	Magnoliopsida	*Sonchus pustulatus*	1
	Magnoliopsida	*Tetraneuris herbacea*	3
	Magnoliopsida	*Alliaria petiolata*	8
	Magnoliopsida	*Boechera fecunda*	5
	Magnoliopsida	*Brassica insularis*	5
	Magnoliopsida	*Braya fernaldii*	1
	Magnoliopsida	*Braya longii*	1
	Magnoliopsida	*Cochlearia bavarica*	1
	Magnoliopsida	*Cochlearia pyrenaica*	1
	Magnoliopsida	*Draba asterophora*	1
	Magnoliopsida	*Lepidium davisii*	8
	Magnoliopsida	*Parolinia glabriuscula*	1
	Magnoliopsida	*Vella pseudocytisus subsp. paui*	9
	Magnoliopsida	*Arenaria grandiflora subsp. bolosii*	1
	Magnoliopsida	*Armeria maritima*	1
	Magnoliopsida	*Armeria merinoi*	3
	Magnoliopsida	*Astrophytum asterias*	3
	Magnoliopsida	*Astrophytum capricorne*	2
	Magnoliopsida	*Astrophytum ornatum*	2
	Magnoliopsida	*Atriplex acanthocarpa*	1
	Magnoliopsida	*Atriplex canescens*	1
	Magnoliopsida	*Echinocactus platyacanthus*	4
	Magnoliopsida	*Eriogonum longifolium var. gnaphalifolium*	8

	Magnoliopsida	*Escobaria robbinsorum*	4
	Magnoliopsida	*Harrisia fragrans*	1
	Magnoliopsida	*Limonium carolinianum*	1
	Magnoliopsida	*Limonium erectum*	1
	Magnoliopsida	*Limonium geronense*	1
	Magnoliopsida	*Limonium malacitanum*	1
	Magnoliopsida	*Mammillaria crucigera*	1
	Magnoliopsida	*Mammillaria dixanthocentron*	1
	Magnoliopsida	*Mammillaria gaumeri*	10
	Magnoliopsida	*Mammillaria hernandezii*	3
	Magnoliopsida	*Mammillaria huitzilopochtli*	1
	Magnoliopsida	*Mammillaria magnimamma*	3
	Magnoliopsida	*Mammillaria napina*	2
	Magnoliopsida	*Mammillaria pectinifera*	1
	Magnoliopsida	*Mammillaria solisioides*	1
	Magnoliopsida	*Mammillaria supertexta*	1
	Magnoliopsida	*Neobuxbaumia macrocephala*	2
	Magnoliopsida	*Neobuxbaumia mezcalaensis*	1
	Magnoliopsida	*Neobuxbaumia polylopha*	1
	Magnoliopsida	*Neobuxbaumia tetetzo*	3
	Magnoliopsida	*Pachycereus pecten-aboriginum*	3
	Magnoliopsida	*Pediocactus bradyi*	26
	Magnoliopsida	*Polygonella basiramia*	2
	Magnoliopsida	*Pterocereus gaumeri*	2
	Magnoliopsida	*Rumex rupestris*	1
	Magnoliopsida	*Saponaria bellidifolia*	3
	Magnoliopsida	*Silene acaulis*	2
	Magnoliopsida	*Silene douglasii var. oraria*	2
	Magnoliopsida	*Silene regia*	1
	Magnoliopsida	*Stenocereus eruca*	1
	Magnoliopsida	*Cornus florida*	1
	Magnoliopsida	*Dipsacus fullonum*	4
	Magnoliopsida	*Nardostachys jatamansi*	1
	Magnoliopsida	*Shorea leprosula*	1
	Magnoliopsida	*Succisa pratensis*	5
	Magnoliopsida	*Ardisia elliptica*	10
	Magnoliopsida	*Grias peruviana*	1
	Magnoliopsida	*Primula elatior*	10
	Magnoliopsida	*Primula farinosa*	4
	Magnoliopsida	*Primula veris*	2
	Magnoliopsida	*Primula vulgaris*	23
	Magnoliopsida	*Pyrrocoma radiata*	5
	Magnoliopsida	*Rhododendron maximum*	2

	Magnoliopsida	*Rhododendron ponticum*	5
	Magnoliopsida	*Sarracenia purpurea*	4
	Magnoliopsida	*Vitaliana primuliflora*	1
	Magnoliopsida	*Acacia bilimekii*	1
	Magnoliopsida	*Anthyllis vulneraria*	24
	Magnoliopsida	*Astragalus peckii*	1
	Magnoliopsida	*Astragalus scaphoides*	18
	Magnoliopsida	*Astragalus tremolsianus*	1
	Magnoliopsida	*Astragalus tyghensis*	6
	Magnoliopsida	*Chamaecrista lineata var. keyensis*	7
	Magnoliopsida	*Cytisus scoparius*	3
	Magnoliopsida	*Lathyrus Vernus*	8
	Magnoliopsida	*Lotus arinagensis*	2
	Magnoliopsida	*Lupinus arboreus*	1
	Magnoliopsida	*Lupinus lepidus*	5
	Magnoliopsida	*Lupinus tidestromii*	3
	Magnoliopsida	*Oxytropis jabalambrensis*	2
	Magnoliopsida	*Prosopis laevigata*	1
	Magnoliopsida	*Tetraberlinia bifoliolata*	1
	Magnoliopsida	*Alnus incana subsp. rugosa*	3
	Magnoliopsida	*Castanea dentata*	19
	Magnoliopsida	*Nothofagus fusca*	1
	Magnoliopsida	*Quercus rugosa*	1
	Magnoliopsida	*Asclepias meadii*	1
	Magnoliopsida	*Gardenia actinocarpa*	2
	Magnoliopsida	*Gentiana pneumonanthe*	1
	Magnoliopsida	*Erodium paularense*	2
	Magnoliopsida	*Geranium sylvaticum*	3
	Magnoliopsida	*Cryptantha flava*	1
	Magnoliopsida	*Dicerandra frutescens*	6
	Magnoliopsida	*Dracocephalum austriacum*	14
	Magnoliopsida	*Echium vulgare*	1
	Magnoliopsida	*Mimulus cardinalis*	1
	Magnoliopsida	*Pinguicula alpina*	1
	Magnoliopsida	*Pinguicula villosa*	1
	Magnoliopsida	*Plantago coronopus*	12
	Magnoliopsida	*Plantago media*	3
	Magnoliopsida	*Ramonda myconi*	6
	Magnoliopsida	*Rosmarinus tomentosus*	3
	Magnoliopsida	*Oxandra asbeckii*	1
	Magnoliopsida	*Anarrhinum fruticosum*	1
	Magnoliopsida	*Antirrhinum subbaeticum*	3
	Magnoliopsida	*Collinsia verna*	1

	Magnoliopsida	*Euphorbia fontqueriana*	1
	Magnoliopsida	*Linum flavum*	4
	Magnoliopsida	*Linum tenuifolium*	4
	Magnoliopsida	*Pedicularis furbishiae*	1
	Magnoliopsida	*Phyllanthus emblica*	1
	Magnoliopsida	*Sapium sebiferum*	6
	Magnoliopsida	*Verbascum fontqueri*	4
	Magnoliopsida	*Viola elatior*	2
	Magnoliopsida	*Viola pumila*	2
	Magnoliopsida	*Daphne rodriguezii*	5
	Magnoliopsida	*Helianthemum juliae*	1
	Magnoliopsida	*Helianthemum polygonoides*	1
	Magnoliopsida	*Helianthemum teneriffae*	1
	Magnoliopsida	*Kosteletzkya pentacarpos*	1
	Magnoliopsida	*Clidemia hirta*	5
	Magnoliopsida	*Oenothera deltoides*	6
	Magnoliopsida	*Verticordia staminosa*	1
	Magnoliopsida	*Vochysia ferruginea*	1
	Magnoliopsida	*Persoonia bargoensis*	3
	Magnoliopsida	*Persoonia glaucescens*	2
	Magnoliopsida	*Actaea cordifolia*	2
	Magnoliopsida	*Actaea elata*	7
	Magnoliopsida	*Actaea spicata*	3
	Magnoliopsida	*Anemone patens*	1
	Magnoliopsida	*Aquilegia chrysantha*	1
	Magnoliopsida	*Aquilegia sp.*	1
	Magnoliopsida	*Dicentra canadensis*	2
	Magnoliopsida	*Ranunculus acris*	1
	Magnoliopsida	*Ranunculus peltatus*	1
	Magnoliopsida	*Sarcocapnos baetica*	1
	Magnoliopsida	*Sarcocapnos pulcherrima*	2
	Magnoliopsida	*Trollius europaeus*	5
	Magnoliopsida	*Trollius laxus*	7
	Magnoliopsida	*Agrimonia eupatoria*	3
	Magnoliopsida	*Geum rivale*	2
	Magnoliopsida	*Horkelia congesta*	1
	Magnoliopsida	*Potentilla anserina*	2
	Magnoliopsida	*Purshia subintegra*	1
	Magnoliopsida	*Rubus praecox*	1
	Magnoliopsida	*Rubus saxatilis*	3
	Magnoliopsida	*Boswellia papyrifera*	1
	Magnoliopsida	*Entandrophragma cylindricum*	1
	Magnoliopsida	*Khaya senegalensis*	1

	Magnoliopsida	*Swietenia macrophylla*	1
	Magnoliopsida	*Saxifraga aizoides*	1
	Magnoliopsida	*Ipomoea leptophylla*	1
	Magnoliopsida	*Ipomopsis aggregata*	1
	Magnoliopsida	*Ipomopsis tenuituba*	1
	Magnoliopsida	*Hypericum cumulicola*	11
	Pinopsida	*Callitris columellaris*	1
	Pinopsida	*Pinus strobus*	2
	Pinopsida	*Taxus floridana*	1

Table S2. Model outputs for the correlations among the components of demographic resilience: compensation, resistance, and recovery time. Median represents the median of the posterior distribution. CI low and high are the lower and higher values of the 95% credible intervals, respectively. Rhat is the ratio of the effective sample size to the overall number of iterations, with values close to one indicating convergence values.

Kingdom	Response	Parameter	Median	CI_low	CI_high	Rhat
**Animals**	Compensation	Intercept	0.10	-2.13	2.33	1.00
		Generation time	0.04	-0.01	0.08	1.00
		Mean reproductive output	0.85	0.78	0.91	1.00
		Generation time: Mean reproductive output	0.06	0.02	0.09	1.00
		Matrix dimension	0.01	-0.02	0.05	1.00
	Resistance	Intercept	-0.13	-2.61	2.52	1.00
		Generation time	-0.09	-0.24	0.06	1.00
		Mean reproductive output	0.42	0.22	0.62	1.00
		Generation time: Mean reproductive output	-0.09	-0.20	0.02	1.00
		Matrix dimension	0.18	0.06	0.30	1.00
	Recovery time	Intercept	0.10	-1.87	2.11	1.00
		Generation time	0.31	0.19	0.43	1.00
		Mean reproductive output	0.33	0.16	0.46	1.00
		Generation time: Mean reproductive output	-0.07	-0.16	0.03	1.00
		Matrix dimension	0.47	0.35	0.58	1.00
**Plants**	Compensation	Intercept	-0.10	-1.48	1.45	1.00
		Generation time	0.05	0.03	0.07	1.00
		Mean reproductive output	0.95	0.93	0.97	1.00
		Generation time: Mean reproductive output	0.04	0.02	0.06	1.00
		Matrix dimension	0.06	0.04	0.09	1.00
	Resistance	Intercept	-0.23	-3.10	2.76	1.00
		Generation time	0.19	0.11	0.27	1.00

		Mean reproductive output	0.41	0.34	0.50	1.00
		Generation time: Mean reproductive output	0.01	-0.06	0.09	1.00
		Matrix dimension	0.04	-0.06	0.14	1.00
	Recovery time	Intercept	0.55	-1.82	2.81	1.00
		Generation time	0.25	0.19	0.32	1.00
		Mean reproductive output	0.04	-0.03	0.11	1.00
		Generation time: Mean reproductive output	0.02	-0.04	0.08	1.00
		Matrix dimension	0.28	0.19	0.36	1.00

Robustness of the resilience in life history correlations

Because the life history traits and components of demographic resilient are derived from the same matrix population models (MPMs) in our study, the observed correlations could have been confounded by inherent mathematical relationships between both outputs. For this reason, we checked the robustness of our analyses by comparing the results from MPMs from natural populations to those from simulated MPMs. We simulated two sets of randomly generated MPMs with and without retrogression (*i.e.,* probability of going back one or more stages in the life cycle of the species). We divided the MPMs with and without retrogression because: (1) the ability to retrogress has been shown to confer a faster recovery time in plants (Salguero-Gómez *et al.* 2016); (2) throughout the plant kingdom retrogression is more common than for animals (Salguero-Gómez *et al.* 2015, 2016); (3) differences in the MPMs structure could result in different life history and resilience estimations (as suggested in Kendall *et al.* 2019).

To generate the random MPMs, the elements $a_{i,j}$ of each $\boldsymbol{A}$ MPM were sampled from known distributions. The MPMs were composed of two demographic processes: reproduction and survival-dependent transition probabilities from a particular stage/size/age j in time t to i in t+1. New individuals recruited into the population in the first stage of the life cycle of the generated MPM, corresponding to the first row of (i=1) $\boldsymbol{A}$. The reproductive output (x) was randomly generated following a Poisson distribution using the function *rpois* from R:

$$p(x) = \lambda^x \exp(-\lambda)/x! \quad (1)$$

where λ was randomly sampled from 1 to 100 using the function *sample.int* from R.

The transition probabilities were randomly sampled using the *runif* function from R, which randomly samples from a uniform distribution:

$$f(x) = 1/(max-min) \quad (2)$$

where *max* and *min* were 0 and 1, respectively, to indicate 0% or 100% transition. For those MPMs with no retrogression, we substituted the upper-triangle (other than the top row) of the matrix by zeros. To ensure that individuals at a given stage/size/age were not surviving over 100% between time steps, the sum of each column of the matrix $\boldsymbol{U}$ (without reproduction) was first constrained to 1 by dividing each matrix element $u_{i,j}$ by the sum of all elements Σu_j. Next, we penalised $u_{i,j}$ by stage-specific survival values. To do so, we created for each MPM a stage-specific survival vector $\boldsymbol{\sigma}$, where each element σ_j was randomly sampled using a uniform distribution ranging between 0 and 1 using the same method described above and multiplying them to each column of $\boldsymbol{U}$. By randomly simulating the matrices from known distributions we remove the biological constraints caused by life history trade-offs or the evolutionary history of the species (Stearns 1992). Therefore, comparing the correlations between the demographic resilience components and the life histories derived from empirical *vs.* random matrices allows us to depict whether they are mathematical artefacts, or due to biological processes.

We then used the resulting MPMs, virtual species and randomly generated ones, to calculate the life history traits and the demographic resilience components, following the same methodology applied in the main manuscript. From the 11,400 simulated matrices, we only used those that were irreducible, primitive, and ergodic (Stott *et al.* 2010b). This selection process resulted in a total of 5,699 randomly

generated MPMs with retrogression, and 5,700 random MPMs without retrogression. Finally, we fitted the same multivariate multilevel Bayesian models used in the main manuscript (see *Statistical analyses*) but using each different set of simulated matrices. We then compared the results with those obtained using the natural populations.

Our results show that most of the correlations among the life history traits and the resilience components are not spurious. The size effects of the models based on natural populations *vs*. simulated MPMs only raise similar correlations between the mean reproductive output and compensation (Fig. S1). This result suggests that the correlation between compensation and mean reproductive output is mathematically constrained. In contrast, the size effects of the other models correlating resilience components and the life history traits show discrepancies among the natural populations *vs.* our simulated MPMs (Fig. S1). These results suggest that our findings are not caused by spurious mathematical correlations between the life history traits and the components of resilience, despite both being calculated from the same MPMs.

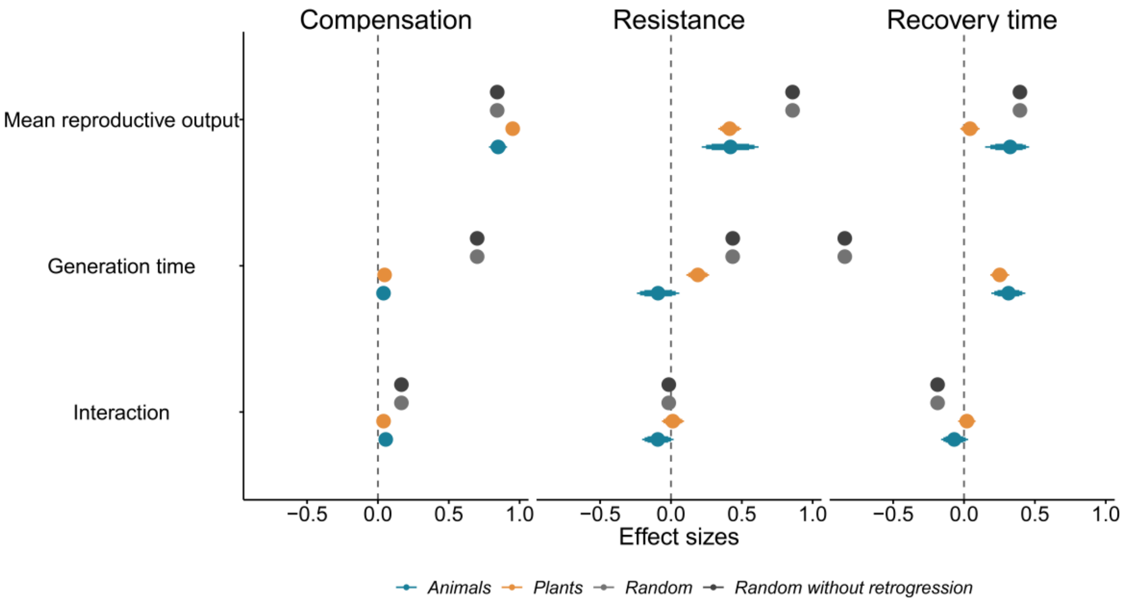

Fig. S1. Compensation is more mathematically constrained than resistance and recovery time in relation to life history traits. Effect sizes of the models evaluating the relationship among the scaled values of resistance, compensation, and recovery time with the scaled values of mean reproductive output, generation time and their interaction. The correlations were performed using 164 natural populations of 76 animal species (blue) and 621 natural populations of 190 plant species (orange) and the 5,699 random MPMs with retrogression (*Random*, light grey), and 5,700 random MPMs without retrogression (*Random without retrogression*, dark grey). The line thickness represents the 95%, 90% and 80% credible intervals of the effect sizes, while the dot represents the mean.

Effects of body size on demographic resilience

To test the influence of the body size of a species on its demographic resilience, we fitted a separated multivariate multilevel Bayesian model for animals and plants. We used compensation, resistance, and recovery time as response variables, and adult body weight (g) for animals or maximum height (m) in plants as fixed effects. We obtained adult body mass (g) data from Myhrvold *et al.* (2015) for mammals birds, reptiles and amphibians, and from FishBase (Froese 2009) for the only elasmobranch species in the study. For terrestrial plants, we utilised maximum

height (m) reported per species in the TRY database (Kattge *et al.* 2011), complemented with information from the Botanical Information and Ecology Network (Maitner *et al.* 2018, BIEN; http://bien.nceas.ucsb.edu/bien/). Not all our species had body dimension information available from these databases or via an online search, and so this limitation reduced our initial sample size. The number of populations of animals decreased from 162 to 144, while for plants it decreased from 748 to 331 populations.

For the models, we used weakly regularising normally-distributed priors for the global intercept and slope:

$$y_{i,j} \sim Normal(\mu_{i,j}, \sigma^2) \quad (3)$$

$$\mu_{i,j} = \beta_0 + \beta_{0i} + \beta_{0j} + \beta_B + \beta_{Bi} + \beta_{Bj} + \beta_D + \beta_{Di} + \beta_{Dj} \quad (4)$$

$$\beta_0 \sim Normal(0,1) \quad (5)$$

$$\beta \sim Normal(0,10) \quad (6)$$

$$\sigma^2 \sim Normal(0,1) \quad (7)$$

where β_0 is the global intercept, β_{0i} and β_{0j} are the population-level and phylogenetic-level departure from β_0, respectively; $y_{i,j}$ is the estimate for compensation, resistance and recovery time for the ith population for the jth phylogenetic distance. β_B and β_D represent the effects of the animal adult body size (g) or plant maximum height (m) and matrix dimension, respectively.

Body dimension influences the components of demographic resilience of species. Both for animals and plants, as the body size increases, compensation

abilities increase (Fig. S2 **a,d**). While this pattern would be expected in plants, where large individuals tend to be highly reproductive and then have a greater ability to compensate for mortality events (Stott *et al.* 2010a; McDonald *et al.* 2016), in most animals larger body sizes are linked to lower reproductive values (Gaillard *et al.* 1989). Resistance is independent of body dimension for both animals and plants, with the slopes of these correlations showing no clear trend (Fig. S2**b,e**). Finally, the animal body size is positively correlated with recovery time (Fig. S2**c**), while plant body height only shows a slightly positive trend (Fig. S2**f**).

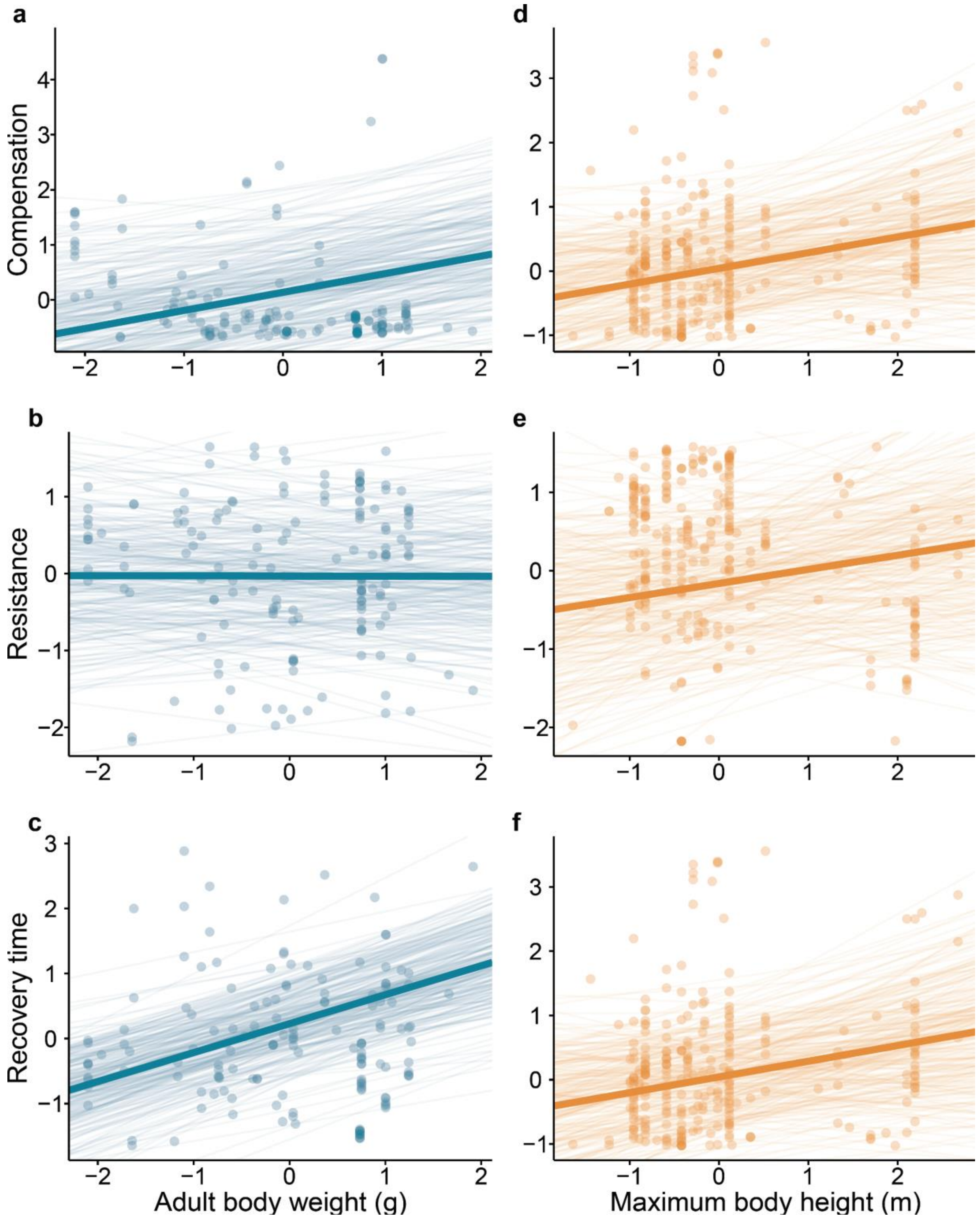

Fig. S2. Correlation between the components of demographic resilience and body dimensions of animals (a, b, and c) and plants (d, e, and f). The correlations between the scaled values of the demographic resilience components of resistance, compensation, and recovery time with the scaled values of adult body weight (g) of 149 natural populations of animals (blue) and 331 plants (orange). Lines

represent the predictions from the multivariate multilevel Bayesian models (Table S2), where thin lines correspond to the predictions drawn from each of the 250 posterior samples of the model, and the thick line represents the mean outcome of the model.

Effects of plant growth form on demographic resilience

To test whether plant growth form shapes demographic resilience, we fitted a multivariate multilevel Bayesian model. We used compensation, resistance, and recovery time as response variables and the life form categorisation of Raunkiær (Raunkiær 1934) as a fixed effect. We classified each plant species according to Raunkiær's growth form scheme (Raunkiær 1934), in which the height of the overwintering shoot apical meristems (SAMs) in relation to ground level determines their life form category. Briefly, the levels used here include: (i) helophyte: SAMs underwater; (ii) geophyte: SAMs underground; (iii) hemicryptophyte: SAMs at ground level; (iv) chamaephyte: SAMs 0-0.25 m height; (v) nanophanerophyte: SAMs 0.25-8 m; (vi) mesophanerophyte: SAMs 8-30 m; and (vii) megaphanerophyte: SAMs >30m. Importantly, Raunkiær's growth form is often associated with the size of the plant, but also accounts for the shape of the plant and its ability to fully regenerate (Raunkiær 1934). Due to a lack of information online for some species, our initial sample size from 748 populations was constrained to 603 populations in this specific test.

We used weakly regularising normally-distributed priors for the global intercept and slope:

$$y_{i,j} \sim Normal(\mu_{i,j}, \sigma^2) \quad (8)$$

$$\mu_{i,j} = \beta_0 + \beta_{0i} + \beta_{0j} + \beta_R + \beta_{Ri} + \beta_{Rj} + \beta_D + \beta_{Di} + \beta_{Dj} \quad (9)$$

$$\beta_0 \sim Normal(0,1) \quad (10)$$

$$\beta \sim Normal(0,10) \quad (11)$$

$$\sigma^2 \sim Normal(0,1) \quad (12)$$

where β_0 is the global intercept, β_{0i} and β_{0j} are the population-level and phylogenetic-level departure from β_0, respectively; $y_{i,j}$ is the estimate for compensation, resistance and recovery time for the *i*th population for the *j*th phylogenetic distance. β_R and β_D represent the effects of the Raunkiær life form and matrix dimension, respectively.

Our examined plant species have different values of compensation, resistance and recovery time depending on their Raunkiær life form (Raunkiær 1934). Still, the overlap among the posterior estimates is considerable (Fig S3), so that species with different growth forms can show similar levels of demographic resilience. Compensation increases from Helophytes to Macrophanerophytes (Fig. S3), indicating that plant species whose shot apical meristems overwinter at a higher distance from the ground have a higher ability to compensate for disturbances. On the contrary, resistance shows no clear patterns across Raunkiær life forms (Fig. S3), suggesting that resistance is independent of the location of SAMs on the plant anatomy. Finally, recovery time displays a concave shape (Fig. S3) from Helophytes to Macrophanerophytes, suggesting that plants with SAMs closer to or further from the ground level have the shortest recovery times post-disturbance. These results are in agreement with previous studies (Stott *et al.* 2010a; McDonald *et al.* 2016),

where trees were reported to have a greater ability to bounce back from disturbances than other plant growth forms due to their higher reproductive outputs. However, we also note a high degree of overlap in the various metrics of demographic resilience across life forms.

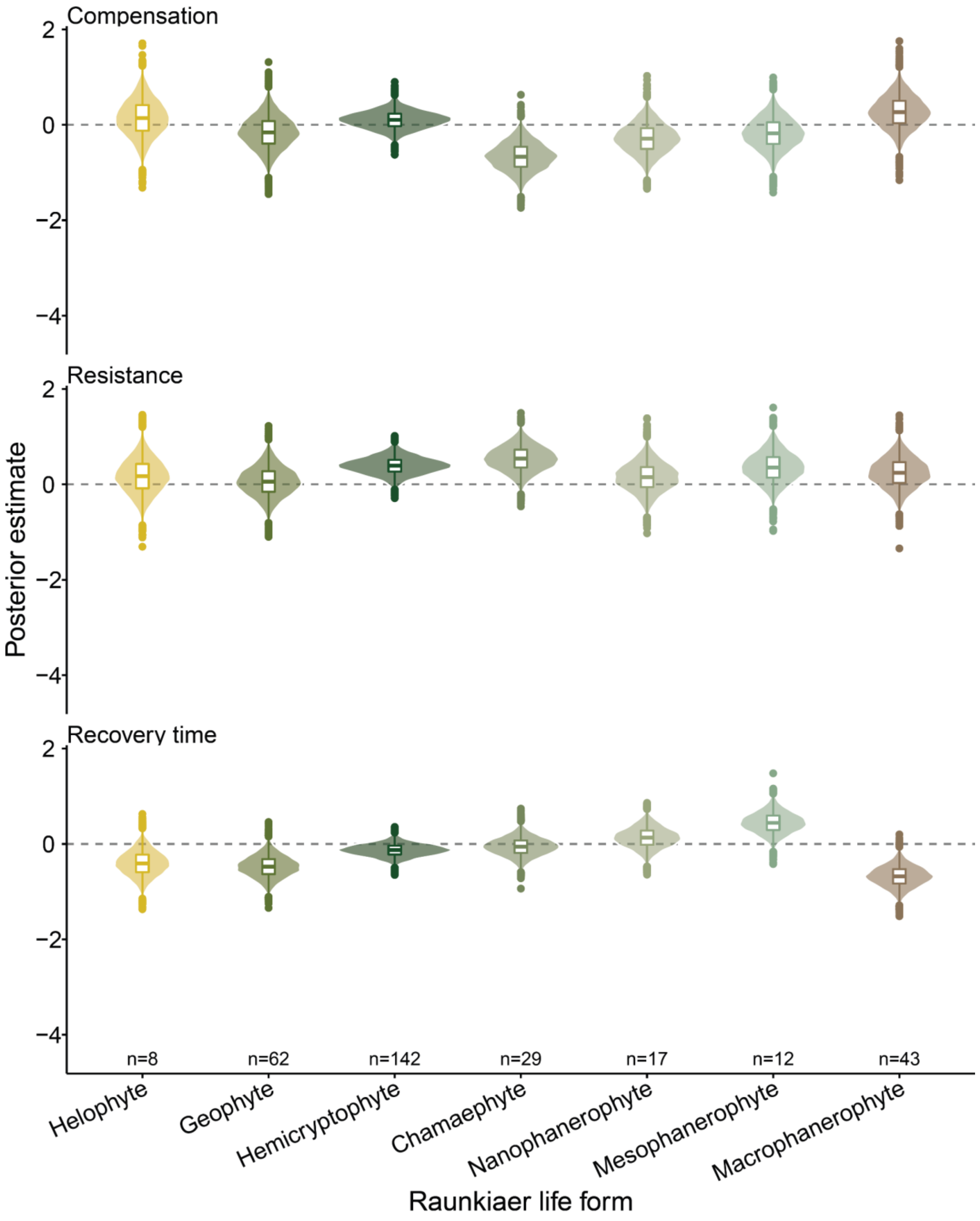

Fig. S3. Demographic resilience components of plants classified according to Raunkiær life forms. We show here the posterior distributions of multivariate multilevel Bayesian models with scaled values of compensation, resistance and recovery time as response variables and the Raunkiær life form classification (Raunkiær 1934). n= indicates the number of populations for which this information was available.

Conservation status

To test for potential correlations between the demographic resilience of the species and their conservation status, we fitted two separate multivariate multilevel Bayesian models for animals and plants. We used compensation, resistance and recovery time as response variables and the conservation status and the conservation status of the studied species as reported on the IUCN Red List (IUCN 2017) as fixed effects. Not all the included species had a conservation status available in the IUCN Red List, reducing our initial sample size from 164 to 155 populations of animals, and from 621 to 212 populations of plants.

For these models we used weakly regularising normally-distributed priors for the global intercept and slope:

$$y_{i,j} \sim Normal(\mu_{i,j}, \sigma^2) \quad (13)$$

$$\mu_{i,j} = \beta_0 + \beta_{0i} + \beta_{0j} + \beta_C + \beta_{Ci} + \beta_{Cj} + \beta_D + \beta_{Di} + \beta_{Dj} \quad (14)$$

$$\beta_0 \sim Normal(0,1) \quad (15)$$

$$\beta \sim Normal(0,10) \quad (16)$$

$$\sigma^2 \sim Normal(0,1) \quad (17)$$

where β_0 is the global intercept, β_{0i} and β_{0j} are the population-level and phylogenetic-level departure from β_0, respectively; $y_{i,j}$ is the estimate for compensation, resistance and recovery time for the ith population for the jth phylogenetic distance. β_C, and β_D represent the effects of the conservation status and matrix dimension, respectively.

There is a high variation in the compensation, resistance and recovery time among the different conservation statuses of the species (Fig. S4). Interestingly, these results suggest that the current conservation status of species is independent of their inherent ability to compensate, resist and recover from disturbances.

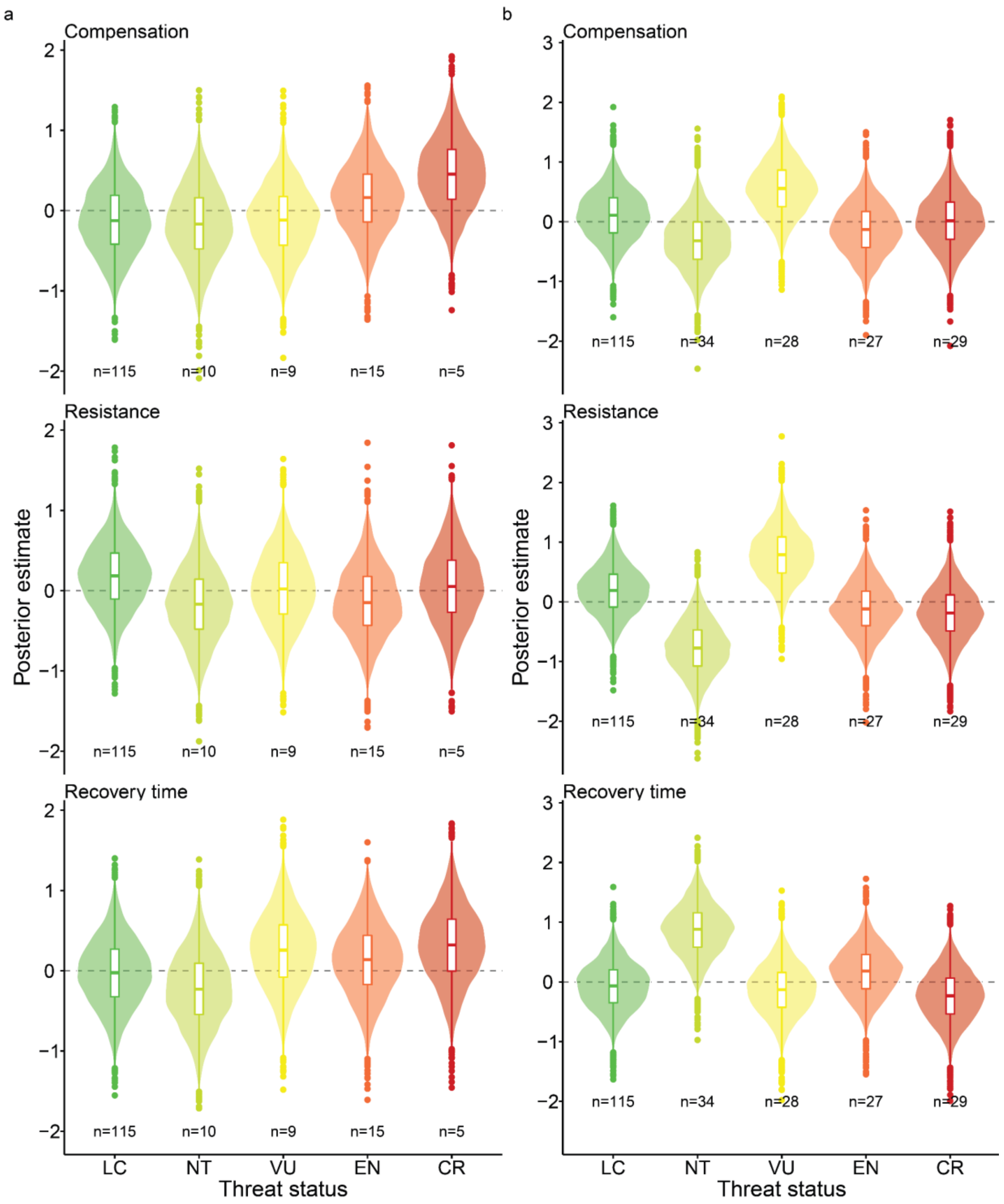

Fig. S4. Demographic resilience components of species against their conservation status in animals (a) and plants (b). We show here the posterior distributions of multivariate multilevel Bayesian models for animals (**a**) and plants (**b**), with scaled values of compensation, resistance and recovery time as response variables and the conservation status of the studied species as reported on the IUCN Red List (IUCN 2017) as fixed effects. LC = Least Concerned, NT = Near Threatened, VU = Vulnerable, EN = Endangered, CR = Critically Endangered. n = indicates the sample size.

References

Froese, R. (2009). FishBase. world wide web electronic publication. *http://www. fishbase. org*.

Gaillard, J.-M., Pontier, D., Allainé, D., Lebreton, J.D., Trouvilliez, J. & Clobert, J. (1989). An analysis of demographic tactics in birds and mammals. *Oikos*, 56, 59–76.

IUCN. (2017). The IUCN Red List of Threatened Species. Version 2017-1.

Kattge, J., Diaz, S., Lavorel, S., Prentice, I.C., Leadley, P., Bönisch, G., *et al.* (2011). TRY–a global database of plant traits. *Global change biology*, 17, 2905–2935.

Kendall, B.E., Fujiwara, M., Diaz-Lopez, J., Schneider, S., Voigt, J. & Wiesner, S. (2019). Persistent problems in the construction of matrix population models. *Ecological modelling*, 406, 33–43.

Maitner, B.S., Boyle, B., Casler, N., Condit, R., Donoghue, J., Durán, S.M., *et al.* (2018). The bien r package: A tool to access the Botanical Information and Ecology Network (BIEN) database. *Methods in Ecology and Evolution*, 9, 373–379.

McDonald, J.L., Stott, I., Townley, S. & Hodgson, D.J. (2016). Transients drive the demographic dynamics of plant populations in variable environments. *Journal of Ecology*, 104, 306–314.

Myhrvold, N.P., Baldridge, E., Chan, B., Sivam, D., Freeman, D.L. & Ernest, S.M. (2015). An amniote life-history database to perform comparative analyses with birds, mammals, and reptiles: Ecological Archives E096-269. *Ecology*, 96, 3109–3109.

Raunkiaer, C. (1934). *The life forms of plants and statistical plant geography; being the collected papers of C. Raunkiaer.* Oxford: Clarendon Press.

Salguero-Gómez, R., Jones, O.R., Archer, C.R., Bein, C., Buhr, H. de, Farack, C., *et al.* (2016). COMADRE: a global data base of animal demography. *Journal of Animal Ecology*, 85, 371–384.

Salguero-Gómez, R., Jones, O.R., Archer, C.R., Buckley, Y.M., Che-Castaldo, J., Caswell, H., *et al.* (2015). The compadre Plant Matrix Database: an open online repository for plant demography. *Journal of Ecology*, 103, 202–218.

Salguero-Gómez, R., Jones, O.R., Jongejans, E., Blomberg, S.P., Hodgson, D.J., Mbeau-Ache, C., *et al.* (2016). Fast–slow continuum and reproductive strategies structure plant life-history variation worldwide. *PNAS*, 113, 230–235.

Stearns, S.C. (1992). *The Evolution of Life Histories*. OUP Oxford.

Stott, I., Franco, M., Carslake, D., Townley, S. & Hodgson, D. (2010a). Boom or bust? A comparative analysis of transient population dynamics in plants. *Journal of Ecology*, 98, 302–311.

Stott, I., Townley, S., Carslake, D. & Hodgson, D.J. (2010b). On reducibility and ergodicity of population projection matrix models. *Methods in Ecology and Evolution*, 1, 242–252.
